# Roles and mechanisms of the m^6^A reader YTHDC1 in biological processes and diseases

**DOI:** 10.1038/s41420-022-01040-2

**Published:** 2022-05-02

**Authors:** Huaqing Yan, Liqi Zhang, Xiaobo Cui, Sinian Zheng, Rubing Li

**Affiliations:** 1 Department of Urology, Ningbo Medical Center Lihuili Hospital, Ningbo, Zhejiang 315000 PR China; 2 Department of Reproductive Medicine, Ningbo First Hospital, Ningbo, Zhejiang 315000 PR China

**Keywords:** Epigenomics, Cancer epigenetics

## Abstract

N6-methyladenosine (m^6^A) is a key area in Epigenetics and has been increasingly focused these years. In the m^6^A process, readers recognize the m^6^A modification on mRNAs or noncoding RNAs and mediate different downstream events. Emerging studies have shown that YTHDC1, an important m^6^A reader, plays a key role in many biological functions and disease progression, especially cancers. Here we summarized the current mechanisms of YTHDC1 in biological functions and diseases and offered guidance for future researches to provide potential strategy for clinical diagnose and therapy.

## Facts

- N6-methyladenosine serves as a crucial area in Epigenetics and has been increasingly focused these years.
- YTHDC1 is a nuclear m^6^A reader and exerts important effects in modulating many biological processes and diseases, especially cancers.
- YTHDC1 can recognize different targets and mediates different RNA fates such as nuclear export, alternative splicing, RNA stabilization and RNA decay.

## Open questions

- Are other new mechanisms of m^6^A reader YTHDC1 still remain undiscovered?
- In the same biological processes or diseases concerning YTHDC1, are different mechanisms or targets exist?
- Can YTHDC1 be selected as a potential biomarker for diagnosis, prognosis or treatment target?
- How can drugs target the specific RNA site according to different mechanisms and precisely modulate RNA fate to reduce side effects?

## Introduction

Epigenetics is defined as heritable variations of gene expression and biological function based on a series of mechanisms without DNA sequence changes. There are many epigenetic modifications reported and researched such as DNA methylation, RNA methylation, genomic imprinting and gene silencing. Among them RNA methylation is a key area in Epigenetics and has been increasingly focused these years. N6-methyladenosine (m^6^A) is the most abundant internal modification in eukaryotic messenger RNAs (mRNAs). The m^6^A RNA modification was first documented as early as in the 1974 but the further researches was not conducted because of technological limitation [1]. Until 2011, the RNA demethylase named the fat mass and obesity associated protein (FTO) was identified and the m^6^A modification process was first determined to be dynamic and invertible, creating a brand new focus in Epigenetics [2].

The m^6^A RNA modification process is achieved by methyltransferase(writers), demethyltransferase (erasers) and reading proteins (readers). METTL3, METTL14, WTAP, RBM15/15B, VIRMA and ZC3H13 are reported to be m^6^A writers catalyzing target RNA methylation which can be reversed by erasers including FTO and ALKBH3/5 [3]. Readers recognize the m^6^A modification on mRNAs or noncoding RNAs and mediate different downstream events. The molecules of YT521-B homology (YTH) domain family, including YTHDF1/2/3 and YTHDC1/2, are the most important and noted readers. Most of these readers have unique mechanisms to perform different biological functions: YTHDF2 is the first reported reader protein which mediates the target mRNA degradation [4]; YTHDF1 augments the translation of m^6^A-modified mRNA and concomitantly affects the overall translational output [5]; YTHDF3 can promote the translation and degradation of mRNA [6]. However, YTHDC1 and YTHDC2 have many functions. Recently emerging studies have shown that YTHDC1 plays a key role in many biological functions and disease progression. Our team focused on the increasing mechanisms of YTHDC1 in biological functions and diseases and offered guidance for future researches to provide potential strategy for clinical diagnose and therapy.

## Role of YTHDC1 in biological functions

### Embryonic development

**Fig. 1:**
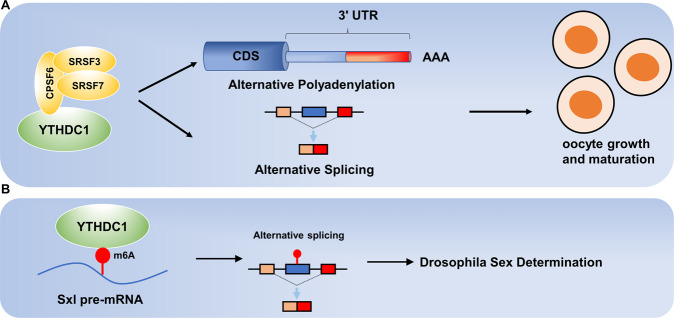
Mechanisms of the m6A reader YTHDC1 in biological processes. **A** YTHDC1 regulates alternative polyadenylation and splicing with the interaction of CPSF6, SRSF3, and SRSF7 to modulate oocyte growth and maturation. **B** YTHDC1 regulates alternative splicing of Sxl pre-mRNA to determine sex in Drosophila.

YTHDC1 plays a key role in embryonic development by a m^6^A-dependent manner. Kasowitz et al. reported that the nuclear m^6^A reader YTHDC1 is essential in male spermatogonia development and female oocyte growth and maturation in mouse [7]. Further research detected widespread alternative splicing defects in YTHDC1-deficient oocytes compared with wild-type oocytes. Apart from the thousands of abnormal alternative splicing events caused by YTHDC1 deficiency, extensive alternative polyadenylation events were found in YTHDC1-deficient oocytes which account for different 3’ UTR length, affecting the mRNA translation and subcellular localization [8]. Co-immunoprecipitation test then was conducted to investigate the underlying mechanisms of YTHDC1 and determined that YTHDC1 is associated with the pre-mRNA 3’ end processing factors CPSF6, SRSF3, and SRSF7 [7](Fig. 1A).

**Fig. 2:**
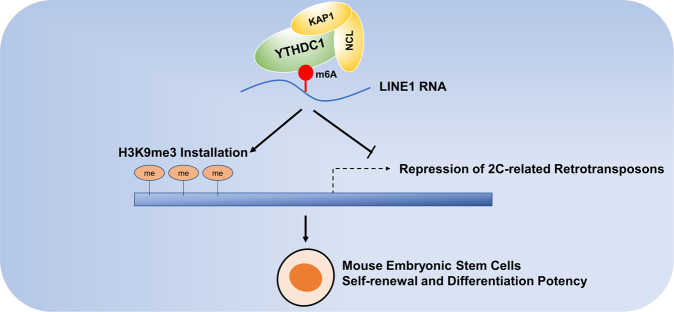
The roles of YTHDC1 in embryonic stem cells. YTHDC1 mediates the self-renewal and represses the 2-cell-like transcription program in mouse embryonic stem cells by recognizing m6A modified LINE1 RNAs on chromatin and facilitating the formation of LINE1-NCL-KAP1 complex to regulate H3K9me3 level on 2-cell-related retrotransposons and repress the 2-cell program.

As early as in 2016, YTHDC1 was reported to modulate neuronal functions and sex determination as a key m^6^A reader in Drosophila [9, 10] (Fig. 1B). In 2020, YTHDC1 was first reported to increase chromatin accessibility and activates transcription by facilitating the decay of chromosome-associated regulatory RNAs (carRNAs) [11]. CarRNAs include promoter-associated-RNA, enhancer RNA and RNA transcribed from transposable elements. Based on this research, Chen et al. found that YTHDC1 mediates the self-renewal and represses the 2-cell-like transcription program in mouse embryonic stem cells by recognizing m^6^A modified LINE1 RNAs on chromatin and facilitating the formation of LINE1-NCL-KAP1 complex to regulate H3K9me3 level on 2-cell-related retrotransposons and repress the 2-cell program, thus determined the role of YTHDC1 in RNA-chromatin cross-talk [12] and revealed that YTHDC1 affects the transcription process and the chromatin accessibility in embryonic development (Fig. 2). Similarly, Liu et al. reported that YTHDC1 binds to the retrotransposons in mouse embryonic stem cells and keeps the repression state of retrotransposons and Dux (the master inducer of the two-cell-like program), which consequently guards the embryonic stem cells’ identity [13]. Moreover, YTHDC1 binds to METTL3 and promotes the association of METTL3 to chromatin, which remains the integrity of embryonic stem cell heterochromatin and silence retroviral elements for mammalian development [14].

### Neuronal development

The m^6^A RNA methylation process is an important mechanism in neuronal mRNA regulation and neuronal development, but the specific mechanisms about YTHDC1 remain to be unveiled [15, 16]. Lence et al. reported that YTHDC1 might be the main mediator of m^6^A function in neuronal functions [9]. Controversially, although the short-term learning and memory ability is dependent of m^6^A pathway, it is YTHDF but not YTHDC1 that mediates the m^6^A process in the short-term learning and memory of Drosophila; Similarly, the cytoplastic m^6^A reader YTHDF1 and YTHDF2 were determined to modulate neuronal development [17, 18]. Future studies are eagerly awaited to reveal the underlying mechanisms between YTHDC1 and neuronal development.

## Role of YTHDC1 in cancers and other diseases

### Lung Cancer

Lung cancer is a common cancer worldwide and ranks the second in cancer incidence with the first cause of cancer death [19]. Surgery is the most appropriate treatment for patients with early-stage non-small-cell lung cancer, while for patients with advanced lung cancer, molecular targeted therapies have yielded encouraging results [20]. The Cancer Genome Atlas(TCGA) database analysis concerning 551 lung squamous cell carcinoma samples showed that YTHDC1 was identified as a prognostic gene (*p* = 0.046, HR = 0.79, 95% CI = 0.62–1.00) in microarray samples using KM Plotter [21]. Additionally, Li et al reported that YTHDC1 was significantly downregulated in lung adenocarcinoma based on the data of 535 lung adenocarcinoma tissues and 347 normal lung tissues from TCGA and the Genotype-Tissue Expression (GTEx) database [22]. Further, Hu et al. retrieved data of lung adenocarcinoma patients from the TCGA and GTEx databases to verify that YTHDC1 was significantly downregulated in lung adenocarcinoma patients and high expression of YTHDC1 was associated with better prognosis [23]. Moreover, data from the STRING database demonstrated that YTHDC1 acted as the hub node of YTHDF1, YTHDF2, and YTHDF3; data from the TIMER2.0 database illustrated that YTHDC1 expression was significantly correlated with infiltration of immune cells [23].

YTHDC1 was verified to modulate circRNA back-splicing to regulate the expression level of target circRNA in a m^6^A dependent manner [24]. In light of the unmentioned mechanism, Liu et al. revealed that in non-small-cell lung cancer cells YTHDC1 regulated m^6^A-mediated circIGF2BP3 genesis by promoting its back-splicing which sponged miR-328-3p as well as miR-3173-5p to upregulate PKP3 expression level [25]. Finally, YTHDC1 mediates the immune escape from CD8^+^ T cell-mediated killing through the downregulation of PD-L1 ubiquitination and following proteasomal degradation by increasing OTUB1 mRNA stability in a PKP3-dependent manner [25].

### Leukemia

Leukemia is the ninth leading cause of cancer incidence in men and the most common cancer in children, accounting for almost 28% of cases [19]. YTHDC1 was revealed to recognize the m^6^A modification on chimeric mRNAs and regulated the nuclear export of the chimeric mRNAs together with long noncoding RNA MALAT1, which promoted the interaction of chimeric mRNAs and m^6^A methyltransferases, in order to modulate acute myelocytic leukemia progression [26]. Chen et al. found that YTHDC1 was the essential m^6^A reader in acute myeloid leukemia and YTHDC1 was required to form nuclear YTHDC1-m^6^A condensates which help YTHDC1 to protect m^6^A-mRNAs from degradation and maintained acute myelocytic leukemia cells survival and the undifferentiated state for leukemia maintenance [27]. Similarly, the oncogenic role of YTHDC1 in acute myeloid leukemia was determined by Sheng et al. who found that YTHDC1 regulated leukemogenesis via a critical regulator of DNA replication MCM4 [28].

### Bladder cancer

Bladder cancer is a common cancer in urinary system and ranks 4_th_ in cancer incidence among men [19]. Through analyzing the RNA sequence data from TCGA, Chen et al. screened the differentially expressed RNA-binding proteins and verified that the bladder cancer patients with higher YTHDC1 expression level tends to have better survival [29]. Then an RNA binding protein-related bladder cancer prognostic model was constructed in which YTHDC1 acted as an independent prognosis-associated RNA-binding protein [29]. In addition, our team found that YTHDC1 was downregulated in bladder cancer cell lines T24 and UM-UC3 and the overexpression of YTHDC1 significantly suppressed the proliferation and metastasis of bladder cancer (data not published). More investigation is expected to unveil the potential mechanism behind the biological functions of YTHDC1 in bladder cancer.

### Hepatocellular carcinoma

Liver cancer is a common cancer worldwide and it is estimated that in 2021 42,230 new cases and 30,230 deaths would be identified in the United States [19]. The YTHDC1 mRNA expression data was downloaded and analyzed from TCGA, ICGC, GSE109211 and GSE78220 by Jiang et al. who revealed that YTHDC1 was significantly overexpressed in hepatocellular carcinoma patients [30]. Further analysis showed that the higher expression of YTHDC1 was statistically related to the poorer survival of hepatocellular carcinoma patients [30]. In another study concerning hepatocellular carcinoma, YTHDC1 favored the cytoplasmic export of m^6^A modified circHPS5 which acted as a miR-370 sponge to downregulate the expression of HMGA2 and thus accelerate the hepatocellular carcinoma tumorigenesis [31].

Hepatitis B virus infections are one of the leading causes of hepatocellular carcinoma oncogenesis. Rao et al. reported that YTHDC1 could bound to the m^6^A-modified circ-ARL3 to promote its back splicing and biogenesis and circ-ARL3 facilitated the Hepatitis B virus-associated hepatocellular carcinoma progression via sponging miR-1305 [32]. Kim et al. indicated that YTHDC1 could recognize m^6^A-methylated HBV transcripts and facilitate their transport to the cytoplasm [33]. Meanwhile YTHDC1 regulated core-associated DNA and subsequent covalently closed circular DNA syntheses and thus affect the viral life cycle [33].

### Colorectal Carcinoma

Colorectal cancer ranks third in cancer incidence and death rate in the United States [19]. Zhuang et al. conducted bioinformatic analysis based on the data of 89 rectal cancer samples and two corresponding adjacent samples from TCGA and reported that the expression level of YTHDC1 was significantly associated with WTAP and METTL14 [34]. Chen et al. revealed that YTHDC1 facilitated the m^6^A modified circNSUN2 cytoplasmic export and the circNSUN2/IGF2BP2/HMGA2 RNA-protein ternary complex in cytoplasm stabilized HMGA2 mRNA to promote colorectal carcinoma liver metastasis progression [35, 36].

### Prostate cancer

Prostate cancer is the most common malignant cancer in elderly men with the highest incidence rate and second death rate in the United States [19]. Luxton et al. demonstrated that the splicing proteins YTHDC1, Sam68 and T-STAR directly interacted with oncogenic metadherin via immunoprecipitation assay and the repression of CD44v5-luc minigene exon inclusion by YTHDC1 could be rescued by metadherin [37]. Further study indicated that C-terminal domain of metadherin is the key construction for the interaction with YTHDC1 and its role of modulating CD44v5 mRNA expression levels. Decreased CD44v5 exon expression level was significantly associated with reduced disease-free survival in prostate cancer patients following radical prostatectomy based on data from TCGA.

### Breast cancer

Breast cancer is the most common cancer in women and ranks second in death rate in the United States in 2021 [19]. Via analyzing the data of 98 triple-negative breast cancer tissues and 114normal breast tissues from TCGA, Wang et al. reported that YTHDC1 was significantly downregulated in triple-negative breast cancer tissues, indicating that YTHDC1 might be a potential tumor suppressor in breast cancer [38]. However, another study on breast cancer cells in vitro achieved the opposite result: YTHDC1 overexpression in MDA-MB-231 cells increased the cell viability and BRCA1/RAD51 expression level [39]. Meanwhile, overexpression of YTHDC1 promoted the resistance to Adriamycin, indicating that YTHDC1 was an oncogene in breast cancer and induced DNA replication and DNA damage repair [39]. Moreover, YTHDC1 was negatively modulated by EMP3, partly through Akt signaling, which was determined a tumor suppressor and inhibited DNA replication, DNA damage repair, chemotherapeutic drug resistance, stem-like properties and Akt-mTOR signaling activation [39]. In the future more researches were expected to comprehensively study the biological function of YTHDC1 in breast cancer to settle the controversy and further investigations on potential mechanisms were needed.

### Glioma

Glioma is the most prevalent and aggressive cancer of the central nervous system and the current therapy includes mainly surgery, radiotherapy and chemotherapy, of which glioblastoma is the most malignant form with poor prognosis [40]. Because of the resistance of traditional treatments and the complexity of the brain, the epigenetic regulator genes have been focused for decades as key biomarkers and treatment targets [41]. Li et al. reported that YTHDC1 bind to the start codon region of SRSF3, SRSF6, and SRSF11 mRNAs and led to the nonsense-mediated mRNA decay of SRSFs in a m^6^A dependent manner, contributing to the glioblastoma phenotype via target mRNA alternative splicing such as BCL-X and NCOR2 [42]. He et al. collected 24 functional single-nucleotide polymorphisms data of 8 m^6^A key genes in 171 glioma child cases and 228 child controls from South China and found that YTHDC1 rs2293595 and YTHDC1 rs3813832 were significantly negative associated with the risk of glioma [43].

### Renal cancer

Renal cancer is one of the most common malignant tumors worldwide. It is estimated that in 2021 approximately 76,080 new cases would be identified in the United States and approximately 80% of the renal cancer are clear cell renal carcinoma [19]. Hagen et al. performed a single-center study by analyzing the tissue samples from patients who underwent radical or partial nephrectomy at the Department of Urology at the University Hospital Bonn and reported that YTHDC1 was significantly downregulated in clear cell renal carcinoma compared to normal tissue in both mRNA and protein level [44]. Moreover, the dysregulated expression of YTHDC1 was associated with worse outcome, demonstrating that YTHDC1 may have the potential role as a biomarker and target for cancer treatment. Similarly, Zhou et al. conducted a retrospective study using TCGA database and found that the downregulation of YTHDC1 was linked with worse clinical characteristics [45].

### Esophageal cancer

The incidence of esophageal cancer ranks 7th worldwide with the 6th mortality rate [46]. In the United States, esophageal cancer ranks 7th in cancer deaths in man with approximately 12,410 deaths in 2021 [19]. However, according to the analysis of 775 patients with esophageal cancer in TCGA, the expression level of YTHDC1 has no significant difference between esophageal cancer tissues and normal tissues [47].

### Pancreatic cancer

Pancreatic cancer is the 7th leading cause of cancer death worldwide and is notorious because of its poor prognosis with almost as many deaths as cases [46]. In the United States pancreatic cancer ranks fourth in cancer death rate and it is estimated that 48,220 deaths would be identified in 2021 [19]. TCGA database analysis showed that YTHDC1 is significantly downregulated and displayed higher genetic mutation frequencies with in-frame deletion as the most frequent mutation type in pancreatic cancer [48]. Hou et al. reported that YTHDC1 promoted the biogenesis of mature miR-30d through m^6^A-mediated modulation of mRNA stability [49]. Moreover, miR-30d could directly target and downregulate the expression level of RUNX1 which act as a transcription factor to regulate the expression of SLC2A1 and HK1, modulating the aerobic glycolysis process and thus miR-30d suppress the viability and metastasis of pancreatic ductal adenocarcinoma as a tumor-suppressive gene [49].

### Endometrial cancer

Endometrial cancer ranks first in gynecologic cancer incidence in the United States, and its incidence is rising [50]. Ma et al. analyzed the data obtained from TCGA database and illustrated that YTHDC1 expression level was lower in endometrial cancer tissues and positively related with immune cell infiltration levels, indicating that YTHDC1 might be a potential biomarker for endometrial cancer diagnosis and prognosis [51].

### Kaposi’s sarcoma

Kaposi’s sarcoma is a multicentric malignancy manifested as lesions varying from several indolent skin lesions to lesions involving one or more organs [52]. The lesions are characterized by proliferation of spindle cells infected with Kaposi’s sarcoma-associated herpesvirus and its lytic replication act as the key role in the development of Kaposi’s sarcoma. YTHDC1, with its associating splicing factors SRSF3 and SRSF10, could bind to the m^6^A sites of RTA pre-mRNA, a key KSHV lytic switch protein, and played an important role in RTA pre-mRNA splicing and viral lytic replication [53]. Interestingly, RTA itself induced m^6^A and enhanced its own pre-mRNA splicing in an m^6^A-dependent manner to modulate Kaposi’s sarcoma-associated herpesvirus lytic gene expression.

### HIV/AIDS

It is estimated that 36.7 million individuals were living with HIV worldwide and approximately 1.8 million new HIV infections occurred while 1 million individuals died from an AIDS-related disease in 2016 [54]. The HIV-1 RNA expression was modulated in a m^6^A dependent manner with YTHDC1 to regulate the alternative splicing of HIV-1 RNAs [55]. Interestingly, YTHDF2 bind to the m^6^A sites on HIV-1 RNAs and resulted in a remarkable increase in the stability of HIV-1 viral RNAs, totally opposite to the previous report about the function of YTHDF2 in cellular mRNAs to destabilize the target mRNA [4, 55].

### Dilated cardiomyopathy

Dilated cardiomyopathy is one of the most prevalent causes of heart failure and the most common indication for heart transplantation worldwide [56]. It is defined by the presence of left ventricular dilatation and contractile dysfunction with genetic mutations involving genes that encode cytoskeletal, sarcomere, and nuclear envelope proteins account for up to 35% of cases [57]. Gao et al. reported that the depletion of YTHDC1 resulted in aberrant splicing of mRNA Titin in a m^6^A-dependent manner and contributes to dilated cardiomyopathy by the promotion of obvious left ventricular chamber enlargement and severe systolic dysfunction and the decreasing of cardiomyocyte contractility and disordered sarcomere arrangement [58].

### Ischemic stroke

Ischemic stroke remains one of the leading causes of disability and death worldwide. Cell injury is an inevitable consequence in the infarct region which exhibits aberrant cell death pathways, thus understanding the potential molecular mechanisms of ischemic stroke is crucial for the future targeted treatment [59]. Zhang et al. found that YTHDC1 expression level is upregulated after ischemia and the overexpression of YTHDC1 protected rats from brain injury through PTEN mRNA degradation to promote AKT phosphorylation [60].

### Diabetic skin

Diabetes is a severe public health threat worldwide, leading to serious to life-threatening complications such as diabetic nephropathy, diabetic retinopathy, diabetic neuropathy and diabetic foot. It is generally accepted that diabetic foot displays significant delays in wound healing of the skin. Exploring the molecular mechanisms of diabetic skin non-healing may help clinicians to find more effective target therapies to promote diabetic skin healing. Liang et al. reported that YTHDC1 was downregulated in keratinocytes under the effects of hyperglycemia and could interact with mRNA SQSTM1, an autophagy receptor, to modulate the autophagy flux in keratinocytes [61]. Moreover, YTHDC1 could bind to ELAVL1/HuR to regulate SQSTM1 mRNA stabilization in the nucleus and knockdown of YTHDC1 increased cell apoptosis rates and delayed wound-healing [61].

## Conclusions and future prospects

**Fig. 3:**
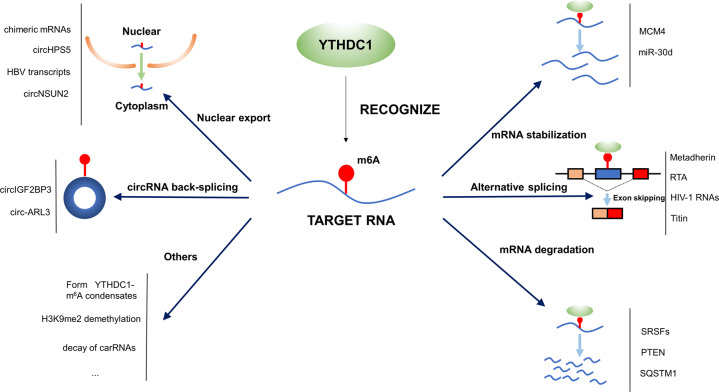
Molecular mechanisms underlying the effect of YTHDC1 on target genes. YTHDC1 recognize the m^6^A marker on target genes and determine the RNA fate, involving nuclear export, alternative splicing, mRNA stabilization, mRNA degradation and others.

In this review, our team outlined the biological functions of m^6^A reader YTHDC1 and more importantly, the potential role of YTHDC1 in cancers and other diseases (Table 1). Meanwhile, a sketch was drawn to illustrate the molecular mechanisms underlying the effect of YTHDC1 on target genes (Fig. 3). The m^6^A modification on RNA can be catalyzed by writers, while the erasers can eliminate the m^6^A modification on RNA. In our review the m^6^A reader YTHDC1 was identified to recognize the m^6^A modification on RNA and affect alternative splicing, mRNA degradation, mRNA stabilization, nuclear export and circRNA back splicing. In addition, YTHDC1 played a critical role in a series of molecular mechanisms. For instance, YTHDC1 was uncovered to promote the formation of the m^6^A-eRNA/YTHDC1 condensate to modulate gene activation [62]; YTHDC1 promoted H3K9me2 demethylation and gene expression [63]; YTHDC1 facilitated the decay of a subset of carRNAs and modulated open chromatin state and downstream transcription [11].

**Table 1:** Roles of YTHDC1 as an m^6^A reader in cancers and other diseases.

Disease	Targets	Molecular mechanism	Cellular function	Ref
Lung Cancer	circIGF2BP3	circRNA back-splicing	Immune response	[25]
Leukemia	chimeric mRNAs	Nuclear export with LncMALAT1	Cancer progression	[26]
	MYC and genes enriched in the MYC signaling pathway	Form nuclear YTHDC1-m^6^A condensates	Maintain cencer cell survival and the undifferentiated state	[27]
	MCM4	mRNA stabilization	Proliferation	[28]
Hepatocellular carcinoma	circHPS5	Nuclear export	Proliferation and migration	[31]
	circ-ARL3	circRNA back splicing	Proliferation	[32]
	Hepatitis B virus transcripts	Nuclear export with FMRP	Viral life cycle	[33]
Colorectal cancer	circNSUN2	Nuclear export	Metastasis	[35]
Prostate cancer	Metadherin	Alternative splicing	NA	[37]
Breast cancer	NA	NA	Proliferation and chemoresistance	[39]
Glioblastoma	SRSF3, SRSF6, and SRSF11	mRNA degradation	Proliferation	[42]
Pancreatic cancer	miR-30d	mRNA stabilization	Proliferation, metastasis and angiogenesis	[49]
Kaposi’s sarcoma	RTA	Alternative splicing	Viral lytic replication	[53]
HIV	HIV-1 RNAs	Alternative splicing	NA	[55]
Dilated cardiomyopathy	Titin	Alternative splicing	Regulating the normal contractile function	[58]
Ischemic stroke	PTEN	mRNA degradation	Neuronal survival	[60]
Diabetic skin	SQSTM1	mRNA degradation	Autophagy	[61]

*NA* Not Applicable.

Meanwhile, emerging evidence also indicated that YTHDC1 is vital in several cellular functions, such as cancer cell proliferation, angiogenesis, chemoresistance and metastasis [31, 32, 39, 49]. Moreover, YTHDC1 could modulate immune response in lung cancer, suggesting that YTHDC1 might have the potential for promoting the therapeutic efficacy of immune treatments [25]. Interestingly, YTHDC1 not only affects biological functions in eukaryotes, but also plays an important role in virus by regulating viral life cycle [33].

In conclusion, our review summarized and provided a currently comprehensive sight for the diverse roles and mechanisms of m^6^A reader YTHDC1 in biological process and diseases. However, the m^6^A process, as the most abundant modification in mRNA, is dynamic and complex. Although dramatic progress has been made in uncovering the function of m^6^A reader YTHDC1, a comprehensive understanding of YTHDC1 and the concerning m^6^A process still remains distant. We anticipate future studies to explore more interesting mechanisms to fulfill a more precise and colorful landscape of YTHDC1. Meanwhile, we expected more researches focusing on the clinical significance of YTHDC1, such as early screening biomarker, prognosis predictor and new options for targeted therapy.
